# Does Social Pension Expansion Relieve Depression and Decrease Medical Costs? Evidence From the Rural Elderly in China

**DOI:** 10.3389/ijph.2022.1604296

**Published:** 2022-03-16

**Authors:** Mi Zhou, Xiaotong Sun, Li Huang

**Affiliations:** College of Economics and Management, Shenyang Agricultural University, Shenyang, China

**Keywords:** depression, rural elderly, pension enrollment, pension income, medical expenditure

## Abstract

**Objectives:** This study was designed to explore the effect of the New Rural Pension Scheme on depressive symptoms or medical costs induced by depression.

**Methods:** We used the Logit, OLS and 2SLS models to explore the impact of the pension on depression and medical costs. We also adopt the method of quantile regression and discontinuity regression to verify the causal relationship between the New Rural Pension Scheme and depression or medical costs induced by depression of the rural elderly.

**Results:** We have found that the New Rural Pension Scheme decreases depressive symptoms of elderly in rural China (OR = 0.90), and the medical costs induced by depressive symptoms by 4.6%. Regression discontinuity results showed that pension significantly reduced the depressive symptoms (depression) and the medical expenditure caused by depressive symptoms (depression) by using parametric and non-parametric methods, and performing a placebo test. The mediating effect results showed that pension may improve mental health by increasing confidence about the future.

**Conclusion:** We demonstrate that the pension significantly decreased both mental health problems and the medical expenses due to depressive symptoms and depression of elderly in rural China. Therefore, our results suggest that the Chinese government should perfect the New Rural Pension Scheme to eliminate barriers to mental health resources, especially for the rural elderly.

## Introduction

### Background

Depression symptoms will be the second leading cause of death after cancer (1). The number of people suffering from depression in China has reached 90 million (2). In recent years, research shows that the detection rate of depression among the elderly in China has reached 33% (3, 4). The study also shows that, as a special group, the rural elderly have more serious depressive symptoms than the urban elderly because of worse self-care ability, poor economic and physical conditions (5). Depression seriously damages the physical and mental health of the elderly in rural China (6, 7). For instance, depressive symptoms increase the incidence rate of acute diseases (such as heart disease and cancer) and chronic diseases (such as high blood sugar, high cholesterol, high blood pressure, and arthritis) among the rural elderly (8). A study conducted in China found that the per annual capita medical cost of adults with depressive status in 2012 was estimated to be 268.8 CNY (or around 41 USD). For the elderly, the cost is as high as 525.6 CNY (or around 81 USD) (9).

Research shows that there is a relationship between health and income (10). The New Rural Pension Scheme is a main source of income for the elderly in rural China. This program was set up in 2008, combining social pooling and personal accounts. Funding comes from individual contributions, collective grants and government subsidies. Although the New Rural Pension Scheme grants some income, the income of the elderly in rural areas is lower than that in urban areas (11). To our knowledge, while previous studies have found a strong relationship between pension income and mental health (12), few studies have demonstrated a causal relationship (13, 14). The findings are inconsistent. For example, previous studies have found that higher pension income leads to higher mortality (15), however, others have found that higher pension income leads to better physical and mental health (16) or lower mortality (17).

In view of these mixed evidence, the purpose of this article is to ascertain whether the New Rural Pension Scheme positively or negatively impacts depressive symptoms and medical costs induced by depression among the rural elderly in China. In pursuing this study object, we estimate the overall impacts of the New Rural Pension Scheme on depressive symptoms and medical spending, and then explore the mechanism by which these effects occur, in particular, confidence about the future among the rural elderly. Pension income may improve mental health by making pension recipients more confident about the future. Therefore, New Rural Pension Scheme can reduce psycho-social stress and negative emotions associated with financial difficulties, as well as increase feelings of control and self-esteem (18, 19).

### Theoretical Frameworks

Under the health economics theory, Mushkin (20) defined “health” as “the component of human capital.” Health economics provides an important economic theoretical basis for solving national physical and mental health problems in key areas of social concern (21). The research of health economics believe that income is an important factor affecting health, and there is a significantly correlation between income and health (22). Although it is still controversial whether the increase of income have a positive or negative effect on health under the theoretical framework of health economics, relevant studies in this field have proved the direct or indirect impact of income on health (23). For example, individuals will feel depressed and frustrated by comparing with other individuals who have better income status. It will be transformed into great psychological pressure and bad behaviors such as drinking and smoking, thus making their health worse (24, 25).

The influencing factors of mental health are extremely complex, affected by different stages of social environment and material economy (26). Studies have found that mental health is related to the physical health (such as heart disease, hypertension, diabetes) and socioeconomic status (such as education level, marital status, personal and family income) of China’s aging population (27–29). The Grossman model of health capital (30, 31) provides a conceptual basis for analyzing the relationship between income and mental health. From Grossman’s framework, pension income for elderly people aged 60 or older may impact mental health through three pathways: (a) Changes in living conditions and entertainment methods; (b) healthcare consumption; and (c) pension income may reduce financial pressure.

Firstly, due to pension income, elderly people who like to live independently may have a healthy psychological state through the pathway (a). This is partly because elderly with pensions are more independent and have a stronger sense of self-actualization. The breakdown of the extended family may reduce family conflict (32). Recent studies on the New Rural Pension Scheme indicate that pension income allows elderly to live more independently in China (33), spending less time caring for grandchildren (34), and with children of elderly more likely to move out to work or even to live away from their hometown (35). Also relevant, prior work finds the New Rural Pension Scheme is associated with the targeted age group hiring others to relieve arduous household chores resulting in increased leisure time (36). These changes in lifestyle are protective factors for mental wellbeing (37).

Secondly, through pathway (b), pensions can improve mental health status of the elderly by reducing the relative expenditures of medical cost. Stigmatization of mental diseases (38, 39), low mental health attention (40) and limited capacity to cure mental diseases are common in China (41). Combined with these factors, the proportion of Chinese patients with mental illness receiving treatment is very low (8%) (42). Therefore, we estimate that health investment pathway (b) is mainly an indirect impact, that is, improving mental health through better nutritional intake and treatment of physical health status. Recent studies on the New Rural Pension Scheme show that pension promotes use of appropriate healthcare services, adherence to recommended treatment plans (34, 36), and nutritional intake (43), with no apparent increase in unhealthy behavior, including smoking and alcohol drinking (43).

Finally, pathway (c) shows that pension income can improve mental health by increasing self-respect and sense of control, as well as reducing psychosocial stress and negative emotions associated with financial difficulties (18, 19).

### New Rural Pension Scheme

“New Rural Pension Scheme” refers to a new established program that combines social pooling and personal account since 2008. It is financed by a combination of personal contribution, collective subsidy, and government subsidy.

The basic pension financed solely by the government is available to all enrollees at age 60 (44). There are five categories of premiums for individual accounts: 100, 200, 300, 400, and 500 CNY (or around 15, 31, 46, 62, and 77 USD) per year per person. While some provinces offer additional higher levels of individual premiums, a majority of participants choose to contribute 100 CNY (or around 15 USD) per year per person, the lowest level of pension premium (45).

## Methods

### Data Source and Sampling

This paper used 2012, 2016 and 2018 data from the China Family Panel Studies (CFPS) to create panel datasets that included the same depression scale (Center for Epidemiologic Studies Depression Scale). The panel dataset contains 2,157 observations of rural elderly over a three-year period, after excluding samples that were missing information (Supplementary Figure S1).

### Measurement

The pivotal part of the questionnaire used in this paper is mainly composed of questions from the Center for Epidemiologic Studies Depression Scale (CES-D). The CES-D scale has been widely extensive used to estimate depressive symptoms in domestic and foreign literatures (46, 47). As shown in Supplementary Table S1, the CES-D scale consisted of 20 items. Scores range from 0 to 60. A CES-D total scores between 20 and 27 represent “depressive symptoms,” and scores of 28 or higher represent “depression” (2). The Chinese version of CES-D scale has been widely used in the existing literature and has passed the examination of reliability and validity in the Chinese population (48, 49).

### Statistical Analyses

The relationship between pension enrollment/pension income and mental health/medical cost induced by mental health can be identified in the following regression. Because mental health is a categorical variable and medical spending induced by mental health is a continuous variable, we used Logit and OLS to perform regression estimation respectively.

To avoid reverse causation and omitted variable bias and obtain unbiased and consistent estimates, we measure community-level monthly pension income and use this variable as an instrument for actual pension enrollment status and pension income. We use the two-stage least-squares (2SLS) computational method to identify our IV estimates.

In order to test the mediating effect of the New Rural Pension Scheme to improve mental health, we use the mediating effect proposed by Wen et al. (50) to test whether the New Rural Pension Scheme improves mental health by increasing individual confidence in the future.

Furthermore, we utilize a similar age discontinuity in the benefit structure of the social pension program to overcome the empirical challenge of endogenous pension receipt. The regression discontinuity (RD) design is a rigorous quasi-experimental approach that can be used to estimate intervention impacts as long as the intervention adopts a continuous measure (force variable) with a clearly defined threshold (cut-off score) to determine who is eligible and who is not (51). RD can both identify causal relationships and mitigate the endogenous problems arising from reverse causality and misspecification (52–55).

## Results

### Descriptive Statistical Analysis

Supplementary Table S2 shows the characteristics of the major variables. The average CES-D score for rural elderly was 14.517, among which 17.43% were depressive symptoms and 9.92% suffered from depression. Depression can create a serious medical burden (2). Medical expenses induced by depressive symptoms (depression) for the rural elderly were 529.732 CNY or around 81 USD (433.307 CNY or around 67 USD) per month. In terms of the New Rural Pension Scheme, 41.63% of rural elderly participated in the pension, and each person in the total sample received an average of 75.714 CNY (or around 12 USD) per month.

### Regression Results

Table 1 Group A lists the key regression results. The baseline regression model indicates that pension enrollment has a significant impact on both the probability of suffering from depression and depressive symptoms for the rural elderly in China. To be specific, the estimation results show that the elderly in rural areas who participate in the New Rural Pension Scheme are less likely to suffer from depressive symptoms (OR = exp(−0.103) = 0.90) and less likely to be depressed (OR = 0.75) than people without pension. These outcomes are in agreement with recent evidence that show that pension enrollment significantly relieves depression (10). Table 1 Group B shows the regression outcomes found by using monthly pension income as an explanatory variable. The outcomes are similar to those from Table 1 Group A.

**TABLE 1:** Effect of New Rural Pension Scheme on mental health in rural China (Logit regression) (China Family Panel Studies, China, 2012, 2016 and 2018)^a^.

Variables	Definition	Group A		Group B	
		Depressive symptoms^b^	Depression^b^	Depressive symptoms^b^	Depression^b^
		1 = Yes	1 = Yes 0 = No	1 = Yes	1 = Yes 0 = No
		0 = No		0 = No	
		(1)	(2)	(3)	(4)
Pension enrollment	1 = Yes; 0 = No	−0.103*** (0.015)	−0.292*** (0.030)		
Pension income	Log			−0.033*** (0.005)	−0.092*** (0.001)
Gender	1 = Male; 0 = Female	−0.268*** (0.007)	−0.362*** (0.027)	−0.273*** (0.008)	−0.380*** (0.029)
Age	Year	−0.014*** (0.002)	−0.003* (0.002)	−0.009*** (0.001)	0.010*** (0.002)
Married^c^	1 = Yes; 0 = No	−0.342*** (0.009)	−0.914*** (0.024)	−0.343*** (0.011)	−0.912*** (0.024)
Widowed^c^	1 = Yes; 0 = No	0.024*** (0.003)	−0.403*** (0.031)	0.013 (0.008)	−0.425*** (0.030)
Single^c^	1 = Yes; 0 = No	0.420*** (0.005)	0.238*** (0.034)	0.409*** (0.002)	0.210*** (0.034)
Education	1 = Illiterate; 2 = Primary school; 3 = Junior high school and above	−0.209*** (0.003)	−0.302*** (0.010)	−0.207*** (0.003)	−0.295*** (0.010)
Income	Log	−0.007*** (0.001)	−0.074*** (0.004)	−0.004*** (0.000)	−0.058*** (0.005)
NRCMS^d^	1 = Yes; 0 = No	0.232*** (0.031)	−0.182 (0.127)	0.235*** (0.030)	−0.176 (0.125)
Family size	Count	−0.004 (0.006)	−0.058*** (0.005)	−0.004 (0.006)	−0.059*** (0.005)
Constant		−0.010 (0.129)	0.285 (0.237)	−0.297*** (0.054)	−0.427* (0.220)
Fixed effect^e^		Yes	Yes	Yes	Yes
Observations		6,471	6,471	6,471	6,471
Number of pid		2,157	2,157	2,157	2,157

Note:

a ***/** Statistically significant at the 1%/5% level. The reported statistics are the marginal effects of the explanatory variables with standard errors shown in parentheses.

b The depressive symptoms/depression group is categorized using CES-D scores (depressive symptoms = CES-D between 20 and 27; depression = CES-D of 28 or higher).

c Marital status includes married, widowed, single, and divorced groups. As the divorced group makes up 0.53% of the sample, we did not add the divorced variable to the regression results and used the divorce sample as a reference category.

d New Rural Cooperative Medical Scheme.

e The regression results control for the regional fixed effect.

Table 2 Group A lists key regression results about the influence of pension enrollment on medical cost induced by depression or depressive symptoms. The results indicate that the rural elderly who take part in the New Rural Pension Scheme expend 4.6%/7.8% less on medical services due to depressive symptoms and depression per year, respectively. Table 2 Group B shows the regression outcomes about the effect of pension income on medical spending due to depressive status. The results are consistent with those from Table 2 Group A.

**TABLE 2:** Effect of New Rural Pension Scheme on medical costs induced by mental health in rural China (Ordinary Least Square regression) (China Family Panel Studies, China, 2012, 2016 and 2018)^a^.

Variables	Definition	Group A		Group B	
		Medical cost induced by depressive symptoms^b^	Medical cost induced by depression^b^	Medical cost induced by depressive symptoms^b^	Medical cost induced by depression^b^
		Log	Log	Log	Log
		(1)	(2)	(3)	(4)
Pension enrollment	1 = Yes; 0 = No	−0.046** (0.015)	−0.078*** (0.012)		
Pension income	Log			−0.010** (0.004)	−0.016*** (0.003)
Control variables^c^		Yes	Yes	Yes	Yes
Fixed effect^d^		Yes	Yes	Yes	Yes
Observations		6,471	6,471	6,471	6,471
Number of pid		2,157	2,157	2,157	2,157

Note:

a ***/**/* Statistically significant at the 1%/5%/10% level. The reported statistics are the marginal effects of the explanatory variables with standard errors shown in parentheses.

b The medical cost induced by depressive symptoms/depression is calculated by the early results of project team in the Supplementary Material “Estimating medical expenditures induced by depressive symptoms and depression” (2).

c Control variables include gender, age, marital status, education, income, New Rural Cooperative Medical Scheme, and family size.

d The regression results control for the regional fixed effect.

### Main Results Based on IV Regression

In the first phase of regression results, this paper tests the relationship between community monthly pension income and personal New Rural Pension Scheme participation rates (Table 3 Group A), including the quantity of pension benefits (Table 3 Group B). Results for the rural elderly indicate that community-level monthly pension income enhances the individual participation rates of pension (OR = 1.15) (Table 3 Group A column (1)) and pension benefits by 73.2 percent points (Table 3 Group B column (1)). These outcomes show that monthly pension income at the county-level is an effective instrumental variable for both New Rural Pension Scheme participation rates and pension income.

**TABLE 3:** Effect of New Rural Pension Scheme on mental health and medical costs induced by mental health in rural China (Instrumental Variable regression) (China Family Panel Studies, China, 2012, 2016 and 2018)^a^.

Group a variables	Definition	First stage (1)		Second stage (2)–(5)		
		Pension enrollment	Depressive symptoms^b^	Depression^b^	Medical cost induced by depressive symptoms^c^	Medical cost induced by depression^c^
		1 = Yes 0 = No	1 = Yes 0 = No	1 = Yes 0 = No	Log	Log
		(1)	(2)	(3)	(4)	(5)
Community-level of monthly pension income^d^		0.139***				
	Log	(0.003)				
Pension enrollment	1 = Yes; 0 = No		−0.141** (0.071)	−0.623*** (0.182)	−0.218** (0.051)	−0.307*** (0.034)
F-statistics		341.000				
*p*-value		0.000				
Group B		Pension income	Depressive symptoms^b^	Depression^b^	Medical cost induced by depressive symptoms^c^	Medical cost induced by depression^c^
		Log	1 = Yes 0 = No	1 = Yes 0 = No	Log	Log
Community-level of monthly pension income^d^		0.732***				
	Log	(0.011)				
Pension income	Log		−0.027** (0.013)	−0.081*** (0.015)	−0.041** (0.010)	−0.057*** (0.006)
F-statistics		519.940				
*p*-value		0.000				
Control variables^e^		Yes	Yes	Yes	Yes	Yes
Fixed effect^f^		Yes	Yes	Yes	Yes	Yes
Observations^g^		6,423	6,423	6,423	6,423	6,423

Note:

a ***/**/* Statistically significant at the 1%/5%/10% level. The reported statistics are the marginal effects of the explanatory variables with standard errors shown in parentheses.

b The depressive symptoms/depression group is categorized using CES-D scores (depressive symptoms = CES-D between 20 and 27; depression = CES-D of 28 or higher).

c The medical cost induced by depressive symptoms/depression is calculated using the early results of the project team in the Supplementary Material “Estimating medical expenditures induced by depressive symptoms and depression” (2).

d The instrumental variables are the community-level of monthly pension income, where a community refers to a rural village in which the respondent lives in.

e Control variables include gender, age, marital status, education, income, New Rural Cooperative Medical Scheme, and family size.

f The regression results control for the regional fixed effect.

g Observations whose residential community contains only one sample individual are dropped, thus the sample size associated with the IV regressions is reduced to 6,423.

In the second phase, the impacts of New Rural Pension Scheme participation rates (Table 3 Group A) and pension benefits (Table 3 Group B) on psychological health and medical costs indicate that pension significantly reduced the degree of depression and the medical expenses due to depressive symptoms and depression. The depressive symptoms of pension recipients is lower than that of individual without pension income in the IV estimation results (OR = 0.87), while OR is 0.54 times about depression. The medical cost due to depressive symptoms/depression is separately 21.8/30.7 percentage points lower compared with pension recipients. The striking difference between the IV regression results and the OLS regression results shows the significance of using the instrumental variable to solve the endogeneity problem of New Rural Pension Scheme. Table 3 Group B shows the regression outcomes about the effect of pension income on medical spending due to depressive status based on IV regression. The results are consistent with those from Table 3 Group A.

### Main Results Based on Mediating Effect Regression

The influencing factors of psychological health and medical spending are complex, including socioeconomic factors and natural circumstances that vary across diverse periods of life (26). Pension enrollment or pension income may improve mental health by increasing confidence about the future. Firstly, we verified the impact of pension enrollment and pension income on future confidence. As is shown in Table 4 (1)–(2), elderly in rural areas who take part in the New Rural Pension Scheme will see an increase in confidence of 0.067 units. In terms of rural elderly pension income, an increase of 1 CNY per month, causes an increase of confidence in the future of 0.019 units. Secondly, we verified the effect of the confidence about the future on mental health and medical costs induced by depressive status. Table 4 shows that each raising in confidence was related to 0.74(OR) times in depressive symptoms and an 22.9% drop in medical expenses induced by depressive symptoms. After considering the mediating effect, the influence of the New Rural Pension Scheme on depressive symptoms and medical costs is still significant, indicating that confidence plays an incomplete mediating effect.

**TABLE 4:** Effect of New Rural Pension Scheme on mental health and medical cost induced by mental health in rural China (Mediating Effect) (China Family Panel Studies, China, 2012, 2016 and 2018)^a^.

Variables	Definition	Confidence about future^b^		Depressive symptoms^c^		Depression^c^		Medical cost induced by depressive symptoms^d^		Medical cost induced by depression^d^	
		1 very unconfident→ 5 very confident		1 = Yes; 0 = No		1 = Yes; 0 = No		Log		Log	
		(1)	(2)	(3)	(4)	(5)	(6)	(7)	(8)	(9)	(10)
Confident about future^b^	1 Very unconfident→5 Very confident			−0.297*** (0.004)	−0.273*** (0.010)	−0.419*** (0.007)	−0.418*** (0.008)	−0.229*** (0.002)	−0.226*** (0.004)	−0.198*** (0.006)	−0.201*** (0.002)
Pension enrollment	1 = Yes; 0 = No	0.067*** (0.030)		−0.079*** (0.015)		−0.263*** (0.014)		−0.016*** (0.003)		−0.026** (0.009)	
Pension income	Log		0.019*** (0.006)		−0.032*** (0.005)		−0.069*** (0.013)		−0.009* (0.004)		−0.004*** (0.001)
Control variables^e^		Yes	Yes	Yes	Yes	Yes	Yes	Yes	Yes	Yes	Yes
Fixed effect^f^		Yes	Yes	Yes	Yes	Yes	Yes	Yes	Yes	Yes	Yes
Observations		6,471	6,471	6,471	6,471	6,471	6,471	6,471	6,471	6,471	6,471
Number of pid		2,157	2,157	2,157	2,157	2,157	2,157	2,157	2,157	2,157	2,157

Note:

a ***/**/* Statistically significant at the 1%/5%/10% level. The reported statistics are the marginal effects of the explanatory variables with standard errors shown in parentheses.

b We used this indicator “How confident are you about your future?” to measure confidence about future of rural elderly. Responses on a scale of 1–5 mean very unconfident to very confident.

c The depressive symptoms/depression group is categorized using CES-D scores (depressive symptoms = CES-D between 20 and 27; depression = CES-D of 28 or higher).

d The medical cost induced by depressive symptoms/depression is calculated using early results of the project team in the Supplementary Material “Estimating medical expenditures induced by depressive symptoms and depression” (2).

e Control variables include gender, age, marital status, education, income, New Rural Cooperative Medical Scheme, and family size.

f The regression results control for the regional fixed effect.

### Heterogeneity Analysis

To discuss the influence of pension enrollment and pension income on mental health or medical cost induced by depression in different characteristics of rural elderly, we divided the rural elderly into different groups by gender, education, chronic disease and economic support from children to discuss the heterogeneous effects. First, Supplementary Table S3 shows heterogeneity analysis based on gender. Compared to men, fewer women smoke or drink, and fewer women attend social events (56). Therefore, pension income has a greater effect on mental health for men. As a more specific example, New Rural Pension Scheme including enrollment and benefits have a significant effect on depressive status and medical spending induced by depression among elderly rural men. These outcomes show that pension income may remove financial pressure of men.

Second, almost two thirds of the elderly among rural areas in the whole CFPS sample are semi-illiterate or illiterate, defined as having not completed primary education. In order to test whether the impacts we found varies with educational background, we divided our sample of rural elderly into three groups, involving samples who are semi-illiterate or illiterate, those who only achieved primary education, and people who attained junior high school. Supplementary Table S4 indicates that pension benefits are the most significant in alleviating depression or medical cost for the illiterate or semi-illiterate sample, which is similar to previous study in the United States (57).

Third, the influence of pension enrollment and income on mental health or medical cost may vary according to the physical health of the rural elderly. Chronic diseases are more common among the elderly (58). Therefore, we examined the heterogeneity of the effect of pensions on depression based on chronic disease. As can be seen from Supplementary Table S5, the negative effect of pension on psychological health was significant among healthy rural elderly. The possible reason is that those with chronic diseases are not getting enough pension income to treat chronic disease.

Finally, it is likely that rural elderly with economic support from their children may benefit more than those without, due to doubled benefits. We divide the rural elderly sample into two sub-samples: those who have economic support from their children, and those who do not. Supplementary Table S6 indicates that rural elderly with who participated in the New Rural Pension Scheme who had economic support from their children will decrease the medical expense induced by depressive symptoms or depression. It may be due to the dual benefits of pension income and financial support for children. However, Supplementary Table S6 shows that those without economic support who receive pension income will show a decrease in depressive symptoms. One possible reason is that pension income plays a greater role in their mental health.

### Robustness Test—Quantile Regression

In this part, a robustness check is described to ensure that the major regression outcomes hold. We consider a quantile regression models by using CES-D score as the explained variable. Supplementary Table S7 indicates that pension enrollment significantly reduced CES-D scores of the rural elderly, as did monthly pension income. The result is significant for any quantile.

### Robustness Test—Regression Discontinuity

This part adopts a method of discontinuity regression to verify the causal relationship between the pension enrollment or income and depression or medical costs due to depression of the rural elderly. New Rural Pension Scheme stipulates these rural residents whose age up to 60 years old and over can obtain basic pension. We take the age 60 as the running variable, it is the discontinuity point of the New Rural Pension Scheme receiving state. The discontinuity plot between age and the participation rate of New Rural Pension Scheme is shown in Supplementary Figure S2, the figure shows that there is a salient span in the participation rate of New Rural Pension Scheme on each side near the discontinuity point (60 years old). The rate of New Rural Pension Scheme on the right-hand side is nearly 0.4 higher than that on the left-hand side.

The specific method of discontinuity regression is as follows: Firstly, this paper uses the CFPS 2012, 2016 and 2018 panel datasets to re-screen sample age. We set the sample boundary to be in the range of [−15, 15] (59) with 60 years as the center (60), and obtain 22,682 observation values between 45 and 75 years old. Secondly. We make linear regression according to the optimal bandwidth calculated by discontinuity regression. The results are as follows:

Table 5 Group A reports RD parametric estimations under the linear model. The New Rural Pension Scheme significantly reduced the depressive symptoms (depression) and the medical expenditure caused by depressive symptoms (depression). To be specific, the New Rural Pension Scheme reduced the incidence of depressive symptoms by 14.2% with 1% significance level.

**TABLE 5:** Effect of New Rural Pension Scheme on depressive symptoms/depression and medical cost induced by depressive symptoms/depression in rural China (Regression Discontinuity) (China Family Panel Studies, China, 2012, 2016 and 2018)^a^.

Group A parametric RD regression variables	Depressive symptoms^b^	Depression^b^	Medical cost induced by depressive symptoms^c^	Medical cost induced by depression^c^
	1 = Yes; 0 = No	1 = Yes; 0 = No	Log	Log
	(1)	(2)	(3)	(4)
RD treatment effect	−0.142*** (0.053)	−0.030** (0.012)	−0.390*** (0.119)	−0.504*** (0.050)
Bandwidth of age	2.01	1.83	2.48	2.75
N^−^|N^+^	1,544|2,370	792|1,595	1,544|2,370	1,544|2,370
Group B	Non-parametric RD regression			
RD treatment effect	−0.083** (0.041)	−0.028*** (0.004)	−1.546** (0.650)	−0.209*** (0.063)
Bandwidth of age	1.63	1.43	1.95	1.99
N^−^|N^+^	792|1,595	792|1,595	792|1,595	792|1,595
Control variables^d^	Yes	Yes	Yes	Yes
Fixed effect^e^	Yes	Yes	Yes	Yes
Observations	18,075	18,075	18,075	18,075

Note:

a ***/**/* Statistically significant at the 1%/5%/10% level. The reported statistics are the RD treatment effects of the explanatory variables with standard errors shown in parentheses.

b The depressive symptoms/depression group is categorized using CES-D scores (depressive symptoms = CES-D between 20 and 27; depression = CES-D of 28 or higher).

c The medical costs induced by depressive symptoms/depression are calculated using the early results of the project team in the Supplementary Material “Estimating medical expenditures induced by depressive symptoms and depression” (2).

d Control variables include gender, age, marital status, education, income, New Rural Cooperative Medical Scheme, and family size.

e The regression results control for the regional fixed effect.

Table 5 Group A mainly used linear regression to inspect the impact of pension on depression and medical expenditure caused by depression of rural elderly. Table 5 Group B will further use the non-parametric method proposed by Austin (61) (based on the estimation of triangular kernel function) to test the robustness.

Table 5 Group B reports RD non-parametric estimations. The New Rural Pension Scheme significantly reduced the depressive symptoms (depression) and the medical expense caused by depressive symptoms (depression). These results are robust on the basis of both parametric estimations and non-parametric estimations.

Supplementary Table S8 shows the findings of a placebo test that suppose the age discontinuity spot at 55 (62) or 65 (63) years old. Clearly, the effect of RD treatment was statistically non-significant. The outcome mainly reports that the skip in the age of 60 years old is reasonable.

## Discussion

Using nationally representative CFPS panel datasets for the years 2012, 2016 and 2018, this article indicated that the New Rural Pension Scheme significantly decreased both mental health problems and the medical expense caused by depressive status for elderly in rural China. Specifically, the depressive symptoms of pension recipients is, on average, 0.9 times compared with that of rural elderly without pension in the baseline estimations, while it is 0.87 times in the IV regressions. The proportion of depression is 0.75 times in pension recipients. Medical costs induced by depressive symptoms of pension recipients are, on average, 4.6% lower than that of non-recipients in the baseline estimation. The quantity of medical expenditures due to depression are 7.8 percentage points lower among pension recipients. This trend is the same for monthly pension income. These results demonstrate that mental health and medical costs associated with depressive symptoms and depression are did an important matter in rural China, and measures should be taken to perfect this pension system and improve mental health among rural elderly.

These outcomes also indicate that the influence is especially great for some sub-groups. One possible explanation of this phenomenon is that pensions play different roles in the lives of different groups. For example, the New Rural Pension Scheme has a greater impact on mental health and medical costs for male than female. Male spend more on daily expenses than female because of drinking and smoking (56). In the issue, the impact of pension income on psychological health is more pronounced in male groups. Less-educated rural elderly may suffer from less depression when they participate in the pension scheme. The New Rural Pension Scheme is a system in which rural elderly participate voluntarily. This scheme can improve a sense of self-actualization (10), and the promotion of spiritual comfort may lead to less serious depression, especially for vulnerable groups. The impact of pension enrollment and pension benefits on psychological health was even greater among rural elderly without chronic diseases. The pension is too small to treat chronic diseases. Rural elderly who received both pensions from the program and economic support from their children have fewer medical costs induced by depressive status. This may be due to the dual benefits of pension income and financial support from children. However, those without economic support from their children also see decreased depressive symptoms and depression when receiving pensions. One possible reason is that pension income plays a greater role in their mental health.

Although no existing researches have comprehensively tested the effect of the New Rural Pension Scheme on medical cost associated with depression among subgroups of Chinese elderly in rural areas, these results about the influence of pensions on mental health are supported by the literature. For example, Chen et al. (10) supplies a new proof of an active causality between pension benefits and psychological health among the elderly, particularly in those who have difficulties in education, finances and health. A study conducted by Ding (64) indicates that old-age pension security is a main factor of subjective well-being among the elderly in rural China, and that the New Rural Pension Scheme may significantly increase the personal life satisfaction. In addition, in spite of low pension levels, the New Rural Pension Scheme indeed alleviate the depressive status of the rural elderly in China (65).

From a policy viewpoint, these findings propose that the government of China should be perfect the New Rural Pension Scheme to eliminate income-caused disorders to psychological health, particularly for the elderly in rural areas. To be specific, the government needs to raise the level of pension benefits gradually and reasonably. The level of pension benefits is the basis for measuring the effect of the New Rural Pension policy, and it is the economic support for the role of the pension scheme. Secondly, the rural elderly should be encouraged to participate in the New Rural Pension Scheme actively. At present, willingness to participate and the level of payment are both low. The government should explore the flexible use of various pension accounts to provide more choices for the rural elderly. Lastly, rural endowment resources should be integrated to establish a sound rural pension system.
