# Supplementary material for: Does Social Pension Expansion Relieve Depression and Decrease Medical Costs? Evidence From the Rural Elderly in China
Source: Int J Public Health. 2022 Mar 16;67:1604296. doi: 10.3389/ijph.2022.1604296 (PMC8966648; doi:10.3389/ijph.2022.1604296)
Supplement: Supplementary file 1 [file DataSheet1.docx]

*Estimating medical expenditures induced by depressive symptoms and depression*

We use the two-part model and counter-factual method to estimate the medical expenditures induced by depressive symptoms and depression. The two-part model divides the medical spending of each person into two processes.

$\Pr(yi>0\left| Xi \right.)=G(\theta1D1i+\theta2D2i+\beta Xi+\mu i)$ (S1)

$yi=\exp(\delta1D1i+\delta2D2i+\gamma Zi)+ei,yi>0，$ (S2)

The explained variable y_i_ refers to the total amount of medical spending in the previous year by person i. The main dummy explanatory variables are whether an individual experienced depressive symptoms (D_1i_) and whether an individual suffered from depression (D_2i_). Variables X_i_ and Z_i_ are a series of personal characteristics including gender, age, marriage status, education, income^[[1]](#footnote-1)^, New Rural Cooperative Medical Scheme (NRCMS)^[[2]](#footnote-2)^, family size and area fixed effect in different equations.

According to the approach used by Hsieh to calculate medical expenditures caused by depression [9], this paper uses the following three-stage method based on the parameters in the two-part model to estimate the count-factual medical costs induced by depressive status among the rural elderly.

$E(yi\left| D1i,D2i,Xi,Zi \right.)=\Pr(yi>0\left| D1i \right.,D2i,Xi)\times E(yi\left| yi>0,D1i,D2i,Zi \right.)$ (S3)

$E(yi\left| D1i=0,Xi,Zi \right.)=\Pr(yi>0\left| D1i \right.=0,Xi)\times E(yi\left| yi>0,D1i=0,Zi \right.)$ (S4)

$E(yi\left| D2i=0,Xi,Zi \right.)=\Pr(yi>0\left| D2i \right.=0,Xi)\times E(yi\left| yi>0,D2i=0,Zi \right.)$ (S5)

$\Delta E1=E(yi\left| D1i,D2i,Xi,Zi \right.)-E(yi\left| D1i=0,Xi,Zi \right.)$ (S6)

$\Delta E2=E(yi\left| D1i,D2i,Xi,Zi \right.)-E(yi\left| D2i=0,Xi,Zi \right.)，$ (S7)

Variable y_i_ refers to the total amount of medical spending in the previous year by person i. The main dummy explanatory variables are whether an individual experienced depressive symptoms (D_1i_) and whether an individual suffered from depression (D_2i_). Variables X_i_ and Z_i_ are a series of personal characteristics including gender, age, marital status, education, income, New Rural Cooperative Medical Scheme, family size and area fixed effect in different equations.

Firstly, we estimate the actual medical expenditure of all subjects by using the fitted values in the equation (S1) and equation (S2) (according to equation (S3)).

Secondly, we set individual mental health status (D_1i_ or D_2i_) to zero to estimate the counter-factual medical expenditure of person i. At the same time, we keep the control variables consistent (according to equation (S4) and equation (S5)).

Finally, we estimate expected individual medical expenditures induced by depressive symptoms and depression (according to equation (S6) and equation (S7)).

The specific results are demonstrated in the author’s previous research [2] and will not be explained in this article because of space limitations.

*Statistical analyses*

The relationship between pension enrollment/pension income and mental health/medical cost induced by mental health can be identified in the following equation (S8):

$Di=\alpha+\theta Pi+\beta Xi+\mu i$ (S8)

The explained variable D_i_ refers to mental health and total amount of medical spending induced by mental health in the previous year by person i. The main explanatory variables are whether an individual reported pension enrollment at the time of the survey and a continuous variable indicating self-reported pension income in the past month. Variable X_i_ represents a series of personal characteristics including gender, age, marital status, education, income, New Rural Cooperative Medical Scheme, family size and area fixed effect. Because mental health is a categorical variable and medical spending induced by mental health is a continuous variable, we used Logit and OLS to perform regression estimation on equation (S8) respectively.

The key empirical challenge in identifying the causal effect of the pension on mental health is that pension income can be endogenous. First, mental health may have a non-negligible impact on pension enrollment and pension income. Second, unobserved factors omitted from the model, such as character traits, life experiences and social network, may affect both mental health and pension enrollment/pension income and can therefore skew results.

To avoid reverse causation and omitted variable bias and obtain unbiased and consistent estimates, we measure community-level monthly pension income and use this variable as an instrument for actual pension enrollment status and pension income.

We use the two-stage least-squares (2SLS) computational method to identify our IV estimates. The corresponding first stage equation of the 2SLS estimations can be identified in the following equation (S9):

$Pi=a+\eta mean\_Pi+\varpi Xi+\varsigma i$ (S9)

P_i_ indicates whether an individual reported pension enrollment at the time of the survey and is a continuous variable indicating self-reported pension income in the past month. The mean_P_i_ is an instrumental variable referring to community-level monthly pension income. Variable X_i_ is a series of personal characteristics including gender, age, marital status, education, income, New Rural Cooperative Medical Scheme, family size and area fixed effect.

In order to test the mediating effect of the New Rural Pension Scheme to improve mental health, we use the mediating effect proposed by Wen et al. [50] to test whether the New Rural Pension Scheme improves mental health by increasing individual confidence in the future. The corresponding first stage equation can be identified in equation (S8). The corresponding second and third stage equation of the mediating effect model can be identified in equation (S10) and equation (S11):

$Ci=\omega+\delta Pi+\gamma Xi+\upsilon i$ (S10)

$Di=\varphi+\theta'Pi+\tau Ci+\rho Xi+\varepsilon i$ (S11)

C_i_ is a mediating variable referring to the degree of confidence in the future. P_i_ indicates whether an individual reported pension enrollment at the time of the survey and is a continuous variable indicating self-reported pension income in the past month. D_i_ refers to mental health and total amount of medical spending induced by mental health in the previous year by person i. Variable X_i_ is a series of personal characteristics including gender, age, marital status, education, income, New Rural Cooperative Medical Scheme, family size and area fixed effect.

Furthermore, we utilize a similar age discontinuity in the benefit structure of the social pension program to overcome the empirical challenge of endogenous pension receipt. The regression discontinuity (RD) design is a rigorous quasi-experimental approach that can be used to estimate intervention impacts as long as the intervention adopts a continuous measure (force variable) with a clearly defined threshold (cut-off score) to determine who is eligible and who is not [51]. RD can both identify causal relationships and mitigate the endogenous problems arising from reverse causality and misspecification [52-55].

Implementation of New Rural Pension Scheme in China can be seen as a natural experiment which meets the above pre-conditions. Below, we briefly describe this method in the context of the current study.

In this study, Age is defined as the force variable with a cut-off score of 60, because only residents aged 60 years or over in the rural area are covered by the New Rural Pension Scheme. For the RD study, the data will be fitted by equation (S12).

$Di=\alpha+\theta Pi+f\left( {age}_{i}-60 \right)+\beta Xi+\mu i$ (S12)

Where D_i_ refers to mental health and total amount of medical spending induced by mental health in the previous year by person i. P_i_ is a binary variable: 1 if individual i receives pension, otherwise 0; θ is the coefficient of interest and measures the extent of pension influencing the health and medical costs; age_i_-60 is the difference between i’s actual age and the cutoff; and f(age_i_-60) is a polynomial form suggested by Calonico [68]; X_i_ is a vector of covariates.

Since a few people below age 60 report receiving pensions because of variations in local policies and implementation [34], we adopt a Fuzzy RD design, which uses a running variable to estimate pension_i_, as shown in equation (S13).

$Pi=\alpha+\theta Ri+f\left( {age}_{i}-60 \right)+\beta Xi+\mu i$ (S13)

Where R_i_ is the running variable, taking the value 1 if individual i is aged 60 or over, and 0 otherwise.


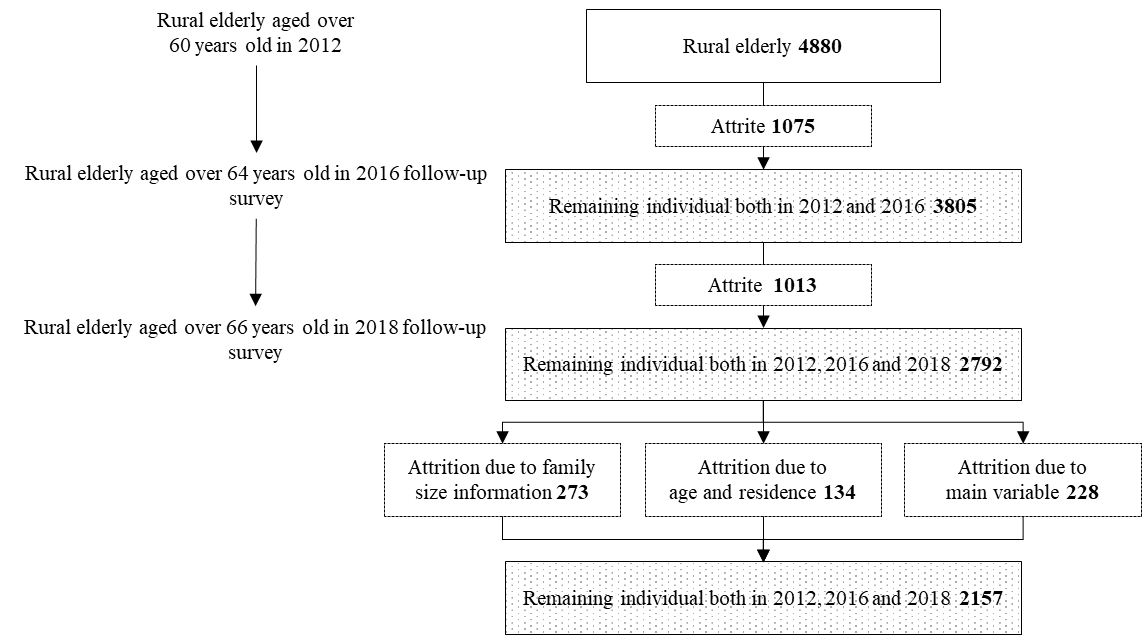


**Figure S1.** The construction of the panel dataset in rural China. Source: China Family Panel Studies, China, 2012, 2016 and 2018. Note: The shaded boxes show the data used in our sample.

**Figure S2.** Regression discontinuity plot in rural China (China Family Panel Studies, China, 2012, 2016 and 2018)

**Table S1.** Center for Epidemiologic Studies Depression Scale (20-item) (China Family Panel Studies, China, 2012, 2016 and 2018)

| Item | During the past week: | Scores ^a^ | | | |
| --- | --- | --- | --- | --- | --- |
|  |  | A | B | C | D |
|  |  |  |  |  |  |
| 1 | I was bothered by things that usually don’t bother me. | 0 | 1 | 2 | 3 |
| 2 | I did not feel like eating; my appetite was poor. | 0 | 1 | 2 | 3 |
| 3 | I felt that I couldn’t shake off the blues even with help from my family or friends. | 0 | 1 | 2 | 3 |
| 4 | I felt I was just as good as other people. | 3 | 2 | 1 | 0 |
| 5 | I had trouble keeping my mind on what I was doing. | 0 | 1 | 2 | 3 |
| 6 | I felt depressed. | 0 | 1 | 2 | 3 |
| 7 | I felt that everything I did was an effort. | 0 | 1 | 2 | 3 |
| 8 | I felt hopeful about the future. | 3 | 2 | 1 | 0 |
| 9 | I thought my life had been a failure. | 0 | 1 | 2 | 3 |
| 10 | I felt fearful. | 0 | 1 | 2 | 3 |
| 11 | My sleep was restless. | 0 | 1 | 2 | 3 |
| 12 | I was happy. | 3 | 2 | 1 | 0 |
| 13 | I talked less than usual. | 0 | 1 | 2 | 3 |
| 14 | I felt lonely. | 0 | 1 | 2 | 3 |
| 15 | People were unfriendly. | 0 | 1 | 2 | 3 |
| 16 | I enjoyed life. | 3 | 2 | 1 | 0 |
| 17 | I had crying spells. | 0 | 1 | 2 | 3 |
| 18 | I felt sad. | 0 | 1 | 2 | 3 |
| 19 | I felt that people disliked me. | 0 | 1 | 2 | 3 |
| 20 | I could not get going. | 0 | 1 | 2 | 3 |

Source: China Family Panel Studies (2012, 2016 and 2018)

Note: ^a^ A means “Rarely or none of the time (less than 1 day)”; B means “Some or a little of the time (1-2 days)”; C means “Occasionally or a moderate amount of time (3-4 days)”; D means “Most or all of the time (5-7 days)”. ^b^ The score is the sum of the 20 questions. Possible range is 0-60.

**Table S2.** Sample summary statistics for key variables about panel dataset in rural China (China Family Panel Studies, China, 2012, 2016 and 2018)

| Variables | Definition | Observations | Mean/% | S.D. | Min | Max |
| --- | --- | --- | --- | --- | --- | --- |
| **Explained variables** |  |  |  |  |  |  |
| CES-D | score | 6,471 | 14.517 | 9.181 | 0 | 52 |
| Depressive symptoms ^a^ | 1=Yes | 1,128 | 17.43% | 0.379 | 0 | 1 |
|  | 0=No | 5,343 | 82.57% | 0.379 | 0 | 1 |
| Depression ^a^ | 1=Yes | 642 | 9.92% | 0.299 | 0 | 1 |
|  | 0=No | 5,829 | 90.08% | 0.299 | 0 | 1 |
| Medical cost induced by depressive symptoms | CNY | 6,471 | 529.732 | 1,113.193 | 0 | 4,268 |
| Medical cost induced by depression | CNY | 6,471 | 433.307 | 936.468 | 0 | 4,455 |
| **Explanatory variables** |  |  |  |  |  |  |
| Pension enrollment | 1=Yes | 2,694 | 41.63% | 0.493 | 0 | 1 |
|  | 0=No | 3,777 | 58.37% | 0.493 | 0 | 1 |
| Monthly pension income | CNY | 6,471 | 75.714 | 225.482 | 0 | 1500 |
| **Instrumental variable** |  |  |  |  |  |  |
| Community-level of monthly pension income ^b^ | CNY | 6,423 ^c^ | 77.830 | 134.397 | 0 | 761.273 |
| **Mediating variable** |  |  |  |  |  |  |
| Confident about future | 1 Very unconfident | 334 | 5.16% | 0.221 | 0 | 1 |
|  | 2 Not confident | 608 | 9.40% | 0.292 | 0 | 1 |
|  | 3 General confident | 1,755 | 27.12% | 0.445 | 0 | 1 |
|  | 4 Confident | 1,555 | 24.03% | 0.427 | 0 | 1 |
|  | 5 Very confident | 2,219 | 34.29% | 0.475 | 0 | 1 |
| **Control variables** |  |  |  |  |  |  |
| Gender | 1=Male | 3,353 | 51.82% | 0.500 | 0 | 1 |
|  | 0=Female | 3,118 | 48.18% | 0.500 | 0 | 1 |
| Age | Year | 6,471 | 68.747 | 5.327 | 60 | 92 |
| Marriage status |  |  |  |  |  |  |
| Married | 1=Yes | 5,297 | 81.86% | 0.385 | 0 | 1 |
|  | 0=No | 1,174 | 18.14% | 0.385 | 0 | 1 |
| Widowed | 1=Yes | 1,059 | 16.37% | 0.370 | 0 | 1 |
|  | 0=No | 5,412 | 83.63% | 0.370 | 0 | 1 |
| Single | 1=Yes | 81 | 1.25% | 0.111 | 0 | 1 |
|  | 0=No | 6,390 | 98.75% | 0.111 | 0 | 1 |
| Divorced | 1=Yes | 34 | 0.53% | 0.072 | 0 | 1 |
|  | 0=No | 6,437 | 99.47% | 0.072 | 0 | 1 |
| Education |  |  |  |  |  |  |
| Illiterate/Semi-literate | 1=Yes | 3,919 | 60.56% | 0.489 | 0 | 1 |
|  | 0=No | 2,552 | 39.44% | 0.489 | 0 | 1 |
| Primary school | 1=Yes | 1,789 | 27.65% | 0.447 | 0 | 1 |
|  | 0=No | 4,682 | 72.35% | 0.447 | 0 | 1 |
| Junior high school and above | 1=Yes | 763 | 11.79% | 0.323 | 0 | 1 |
|  | 0=No | 5,708 | 88.21% | 0.323 | 0 | 1 |
| Income | CNY | 6,471 | 2,459.046 | 7,099.352 | 0 | 120000 |
| New Rural Cooperative Medical Scheme | 1=Yes | 5,932 | 91.67% | 0.276 | 0 | 1 |
|  | 0=No | 539 | 8.33% | 0.276 | 0 | 1 |
| Family size | Number of family members | 6,471 | 3.907 | 2.166 | 1 | 15 |

Source: China Family Panel Studies (2012, 2016 and 2018). Note: ^a^ The Depressive symptoms group and depression group are categorized using CES-D scores (depressive symptoms= CES-D between 20 and 27; depression= CES-D of 28 or higher); ^b^ The instrumental variables are the community-level of monthly pension income, where a community refers to the village in which the respondent lives; ^c^ Observations whose residential community contains only one sample individual are dropped, thus the sample size associated with the IV regressions is reduced to 6,423.

**Table S3.** Heterogeneity analysis based on gender in rural China (China Family Panel Studies, China, 2012, 2016 and 2018)

| Variables | Definition | Depressive symptoms ^b^ | | Depression ^b^ | | Medical cost induced  by depressive symptoms ^c^ | | Medical cost induced  by depression ^c^ | |
| --- | --- | --- | --- | --- | --- | --- | --- | --- | --- |
|  |  | 1=Yes; 0=No | | 1=Yes; 0=No | | Log | | Log | |
|  |  | (1) | (2) | (3) | (4) | (5) | (6) | (7) | (8) |
| Pension enrollment | 1=Yes; 0=No | -0.237*** | -0.022 | -0.384*** | -0.264 | -0.099** | 0.076 | -0.052** | -0.035 |
|  |  | (0.062) | (0.097) | (0.025) | (0.173) | (0.004) | (0.086) | (0.017) | (0.081) |
| Monthly pension income | Log | -0.059*** | -0.005 | -0.102*** | -0.061 | -0.027* | 0.022 | -0.018*** | -0.015 |
|  |  | (0.014) | (0.023) | (0.024) | (0.039) | (0.004) | (0.018) | (0.004) | (0.016) |
| Gender | 1=Male; 0=Female | Male | Female | Male | Female | Male | Female | Male | Female |
| Control variables ^d^ |  | Yes | Yes | Yes | Yes | Yes | Yes | Yes | Yes |
| Fixed effect ^e^ |  | Yes | Yes | Yes | Yes | Yes | Yes | Yes | Yes |
| Observations |  | 3,353 | 3,118 | 3,353 | 3,118 | 3,353 | 3,118 | 3,353 | 3,118 |
| Number of pid |  | 1,117 | 1,040 | 1,117 | 1,040 | 1,117 | 1,040 | 1,117 | 1,040 |

Note: ^a^ ***/**/* Statistically significant at the 1%/5%/10% level; ^b^ The depressive symptoms / depression group is categorized using CES-D scores (depressive symptoms= CES-D between 20 and 27; depression= CES-D of 28 or higher); ^c^ The medical costs induced by depressive symptoms / depression are calculated using the early results of the project team [2]; ^d^ Control variables include age, marital status, education, income, New Rural Cooperative Medical Scheme, and family size; ^e^ The regression results control for the regional fixed effect; ^f^ The reported statistics are the marginal effects of the explanatory variables with standard errors shown in parentheses.

**Table S4.** Heterogeneity analysis based on education in rural China (China Family Panel Studies, China, 2012, 2016 and 2018)

| Variables | Definition | Depressive symptoms ^b^ | | | Depression ^b^ | | | Medical cost induced by  depressive symptoms ^c^ | | | Medical cost induced  by depression ^c^ | | |
| --- | --- | --- | --- | --- | --- | --- | --- | --- | --- | --- | --- | --- | --- |
|  |  | 1=Yes; 0=No | | | 1=Yes; 0=No | | | Log | | | Log | | |
|  |  | (1) | (2) | (3) | (4) | (5) | (6) | (7) | (8) | (9) | (10) | (11) | (12) |
| Pension enrollment | 1=Yes; 0=No | -0.114*** | -0.071 | -0.252 | -0.331*** | -0.406 | -0.690 | -0.078*** | 0.149 | 0.054 | -0.169** | 0.158 | 0.143 |
|  |  | (0.040) | (0.166) | (0.283) | (0.013) | (0.317) | (0.583) | (0.013) | (0.111) | (0.164) | (0.013) | (0.106) | (0.163) |
| Monthly pension income | Log | -0.030*** | -0.034 | -0.034 | -0.076*** | -0.100 | -0.209 | -0.018*** | 0.020 | 0.022 | -0.029* | 0.023 | 0.027 |
|  |  | (0.003) | (0.036) | (0.058) | (0.015) | (0.070) | (0.128) | (0.003) | (0.023) | (0.033) | (0.003) | (0.022) | (0.033) |
| Education 1= Illiterate; 2= Primary; 3= Junior | | Illiterate | Primary | Junior | Illiterate | Primary | Junior | Illiterate | Primary | Junior | Illiterate | Primary | Junior |
| Control variables ^d^ |  | Yes | Yes | Yes | Yes | Yes | Yes | Yes | Yes | Yes | Yes | Yes | Yes |
| Fixed effect ^e^ |  | Yes | Yes | Yes | Yes | Yes | Yes | Yes | Yes | Yes | Yes | Yes | Yes |
| Observations |  | 3,919 | 1,789 | 763 | 3,919 | 1,789 | 763 | 3,919 | 1,789 | 763 | 3,919 | 1,789 | 763 |
| Number of pid |  | 1,282 | 618 | 257 | 1,282 | 618 | 257 | 1,282 | 618 | 257 | 1,282 | 618 | 257 |

Note: ^a^ ***/**/* Statistically significant at the 1%/5%/10% level; ^b^ The depressive symptoms / depression group is categorized using CES-D scores (depressive symptoms= CES-D between 20 and 27; depression= CES-D of 28 or higher); ^c^ The medical costs induced by depressive symptoms / depression are calculated using the early results of the project team [2]; ^d^ Control variables include gender, age, marital status, income, New Rural Cooperative Medical Scheme, and family size; ^e^ The regression results control for the regional fixed effect; ^f^ The reported statistics are the marginal effects of the explanatory variables with standard errors shown in parentheses.

**Table S5.** Heterogeneity analysis based on chronic disease in rural China (China Family Panel Studies, China, 2012, 2016 and 2018)

| Variables | Definition | Depressive symptoms ^b^ | | Depression ^b^ | | Medical cost induced  by depressive symptoms ^c^ | | Medical cost induced  by depression ^c^ | |
| --- | --- | --- | --- | --- | --- | --- | --- | --- | --- |
|  |  | 1=Yes; 0=No | | 1=Yes; 0=No | | Log | | Log | |
|  |  | (1) | (2) | (3) | (4) | (5) | (6) | (7) | (8) |
| Pension enrollment | 1=Yes; 0=No | 0.077 | -0.208*** | -0.389 | -0.378*** | 0.013 | -0.052** | -0.165 | -0.076*** |
|  |  | (0.133) | (0.034) | (0.257) | (0.034) | (0.100) | (0.018) | (0.102) | (0.015) |
| Monthly pension income | Log | 0.004 | -0.049*** | -0.077 | -0.100*** | 0.008 | -0.014** | -0.023 | -0.011** |
|  |  | (0.028) | (0.006) | (0.058) | (0.004) | (0.021) | (0.004) | (0.018) | (0.004) |
| Chronic disease | 1=Yes; 0=No | Yes | No | Yes | No | Yes | No | Yes | No |
| Control variables ^d^ |  | Yes | Yes | Yes | Yes | Yes | Yes | Yes | Yes |
| Fixed effect ^e^ |  | Yes | Yes | Yes | Yes | Yes | Yes | Yes | Yes |
| Observations |  | 1,652 | 4,819 | 1,652 | 4,819 | 1,652 | 4,819 | 1,652 | 4,819 |
| Number of pid |  | 1,110 | 1,047 | 1,110 | 1,047 | 1,110 | 1,047 | 1,110 | 1,047 |

Note: ^a^ ***/**/* Statistically significant at the 1%/5%/10% level; ^b^ The depressive symptoms / depression group is categorized using CES-D scores (depressive symptoms= CES-D between 20 and 27; depression= CES-D of 28 or higher); ^c^ The medical costs induced by depressive symptoms / depression are calculated using the early results of the project team [2]; ^d^ Control variables include gender, age, marital status, education, income, New Rural Cooperative Medical Scheme, and family size; ^e^ The regression results control for the regional fixed effect; ^f^ The reported statistics are the marginal effects of the explanatory variables with standard errors shown in parentheses.

**Table S6.** Heterogeneity analysis based on children’s economic support in rural China (China Family Panel Studies, China, 2012, 2016 and 2018)

| Variables | Definition | Depressive symptoms ^b^ | | Depression ^b^ | | Medical cost induced  by depressive symptoms ^c^ | | Medical cost induced  by depression ^c^ | |
| --- | --- | --- | --- | --- | --- | --- | --- | --- | --- |
|  |  | 1=Yes; 0=No | | 1=Yes; 0=No | | Log | | Log | |
|  |  | (1) | (2) | (3) | (4) | (5) | (6) | (7) | (8) |
| Pension enrollment | 1=Yes; 0=No | 0.009 | -0.219*** | -0.246 | -0.282*** | -0.088*** | 0.043 | -0.178*** | 0.107 |
|  |  | (0.111) | (0.033) | (0.152) | (0.015) | (0.003) | (0.030) | (0.004) | (0.087) |
| Monthly pension income | Log | -0.013 | -0.046*** | -0.057 | -0.072*** | -0.006*** | 0.002 | -0.020* | 0.004 |
|  |  | (0.022) | (0.006) | (0.035) | (0.000) | (0.001) | (0.019) | (0.003) | (0.018) |
| Economic support from children | 1=Yes; 0=No | Yes | No | Yes | No | Yes | No | Yes | No |
| Control variables ^d^ |  | Yes | Yes | Yes | Yes | Yes | Yes | Yes | Yes |
| Fixed effect ^e^ |  | Yes | Yes | Yes | Yes | Yes | Yes | Yes | Yes |
| Observations |  | 3,540 | 2,931 | 3,540 | 2,931 | 3,540 | 2,931 | 3,540 | 2,931 |
| Number of pid |  | 1,773 | 1,655 | 1,773 | 1,655 | 1,773 | 1,655 | 1,773 | 1,655 |

Note: ^a^ ***/**/* Statistically significant at the 1%/5%/10% level; ^b^ The depressive symptoms / depression group is categorized using CES-D scores (depressive symptoms= CES-D between 20 and 27; depression= CES-D of 28 or higher); ^c^ The medical costs induced by depressive symptoms / depression are calculated using the early results of the project team [2]; ^d^ Control variables include gender, age, marital status, education, income, New Rural Cooperative Medical Scheme, family size, and relationship with child. Close relationships with children significantly reduced depressive status and medical cost among rural elderly. Due to space limitations, the regression results are not explained in the main text. Those who are interested can ask for them from the author; ^e^ The regression results control for the regional fixed effect; ^f^ The reported statistics are the marginal effects of the explanatory variables with standard errors shown in parentheses.

**Table S7.** Robustness test based on Center for Epidemiologic Studies Depression Scale in rural China (Quantile regression) (China Family Panel Studies, China, 2012, 2016 and 2018)

| Variables | Definition | CES-D score | | | | |
| --- | --- | --- | --- | --- | --- | --- |
|  |  | 10th quantile | 25th quantile | 50th quantile | 75th quantile | 90th quantile |
|  |  | (1) | (2) | (3) | (4) | (5) |
| Pension enrollment | 1=Yes; 0=No | -0.303* | -0.697*** | -0.840*** | -1.298*** | -1.429** |
|  |  | (0.166) | (0.264) | (0.322) | (0.448) | (0.678) |
| Monthly pension income | Log | -0.086* | -0.259*** | -0.314*** | -0.431*** | -0.338** |
|  |  | (0.049) | (0.057) | (0.073) | (0.102) | (0.154) |
| Control variables ^b^ |  | Yes | Yes | Yes | Yes | Yes |
| Fixed effect ^c^ |  | Yes | Yes | Yes | Yes | Yes |
| Observations |  | 6,471 | 6,471 | 6,471 | 6,471 | 6,471 |

Note: ^a^ ***/* Statistically significant at the 1%/10% level; ^b^ Control variables include gender, age, marital status, education, income, New Rural Cooperative Medical Scheme, and family size. ^c^ The regression results control for the regional fixed effect; ^d^ The reported statistics are the marginal effects of the explanatory variables with standard errors shown in parentheses.

**Table S8** Placebo test of regression discontinuity in rural China (China Family Panel Studies, China, 2012, 2016 and 2018)

| Variables | Depressive symptoms ^b^ | Depression ^b^ | Medical cost induced  by depressive symptoms ^c^ | Medical cost induced  by depression ^c^ | Depressive symptoms ^b^ | Depression ^b^ | Medical cost induced  by depressive symptoms ^c^ | Medical cost induced  by depression ^c^ |
| --- | --- | --- | --- | --- | --- | --- | --- | --- |
|  | 1=Yes; 0=No | 1=Yes; 0=No | Log | Log | 1=Yes; 0=No | 1=Yes; 0=No | Log | Log |
|  | Bandwidth of age: 55 | | | | Bandwidth of age: 65 | | | |
|  | (1) | (2) | (3) | (4) | (5) | (6) | (7) | (8) |
| RD treatment effect | 0.237 | -0.046 | -2.819 | -2.091 | -1.261 | 2.148 | -31.968 | 20.502 |
|  | (0.420) | (0.170) | (2.476) | (1.567) | (2.416) | (5.241) | (285.633) | (27.647) |
| Bandwidth of age | 1.59 | 1.61 | 3.04 | 3.12 | 1.69 | 1.78 | 2.92 | 2.22 |
| Control variables ^d^ | Yes | Yes | Yes | Yes | Yes | Yes | Yes | Yes |
| Fixed effect ^e^ | Yes | Yes | Yes | Yes | Yes | Yes | Yes | Yes |
| N^-^\|N^+^ | 936\|1,680 | 936\|1,680 | 2,880\|3,063 | 2,880\|3,063 |  |  |  |  |
| N^-^\|N^+^ |  |  |  |  | 786\|1,337 | 786\|1,337 | 1,591\|1,892 | 1,591\|1,892 |
| Observations | 18,075 | 18,075 | 18,075 | 18,075 | 18,075 | 18,075 | 18,075 | 18,075 |

Note: ^a^ ***/**/* Statistically significant at the 1%/5%/10% level; ^b^ The depressive symptoms / depression group is categorized using CES-D scores (depressive symptoms= CES-D between 20 and 27; depression= CES-D of 28 or higher); ^c^ The medical costs induced by depressive symptoms / depression are calculated using the early results of the project team [2]; ^d^ Control variables include gender, age, marital status, education, income, New Rural Cooperative Medical Scheme, and family size; ^e^ The regression results control for the regional fixed effect; ^f^ The reported statistics are the RD treatment effects of the explanatory variables with standard errors shown in parentheses.

References

66. Sun, M., Shen, J. J., Li, C., Cochran, C., & Hao, M. (2016). Effects of China’s New Rural Cooperative Medical Scheme on reducing medical impoverishment in rural Yanbian: An alternative approach. *BMC Health Services Research,* *16*, 422. Doi: 10.1186/s12913-016-1660-7

67. Yi, H., Zhang, J., Ma, C., & Ma, S. (2016). Utilization of the NCMS and its association with expenditures: observations from rural Fujian, China. *Public Health,* *130*, 84-86. Doi: 10.1016/j.puhe.2015.05.003

68. Calonico, S., Cattaneo, M. D., & Titiunik, R. (2015). Rdrobust: An R Package for Robust Nonparametric Inference in Regression-Discontinuity Designs. *R Journal, 7,* 38-51. Doi: 10.32614/RJ-2015-004

1. Income refers to self-employment income or wage income earned by working. [↑](#footnote-ref-1)
2. The New Rural Cooperative Medical Scheme is a basic health insurance scheme offered and operated by the central government. It aims to safeguard rural people’s access to basic health services and alleviate the financial burden caused by sickness and poverty with a focus on inpatient services and catastrophic outpatient services [66, 67]. [↑](#footnote-ref-2)
